# The association of employment status with ideal cardiovascular health factors and behaviors among Hispanic/Latino adults: Findings from the Hispanic Community Health Study/Study of Latinos (HCHS/SOL)

**DOI:** 10.1371/journal.pone.0207652

**Published:** 2018-11-27

**Authors:** Mayra L. Estrella, Natalya I. Rosenberg, Ramon A. Durazo-Arvizu, Hector M. Gonzalez, Matthew S. Loop, Richard H. Singer, James P. Lash, Sheila F. Castañeda, Krista M. Perreira, Kamal Eldeirawi, Martha L. Daviglus

**Affiliations:** 1 Institute for Minority Health Research, College of Medicine, University of Illinois at Chicago, Chicago, IL, United States of America; 2 Department of Public Health Sciences, Loyola University Medical Center, Maywood, IL, United States of America; 3 Department of Neurosciences, University of California San Diego, San Diego, CA, United States of America; 4 Department of Biostatistics, University of North Carolina at Chapel Hill, Chapel Hill, NC, United States of America; 5 Department of Public Health Sciences, University of Miami Miller School of Medicine, Miami, FL, United States of America; 6 Division of Nephrology, College of Medicine, University of Illinois at Chicago, Chicago, IL, United States of America; 7 Graduate School of Public Health, San Diego State University, San Diego, CA, United States of America; 8 Department of Social Medicine, School of Medicine, University of North Carolina at Chapel Hill, Chapel Hill, NC, United States of America; 9 Department of Health Systems Science, College of Nursing, University of Illinois at Chicago, Chicago, IL, United States of America; Universidad Miguel Hernandez de Elche, SPAIN

## Abstract

**Background:**

The American Heart Association’s 2020 Impact Goals propose to improve cardiovascular health (CVH) and reduce deaths from cardiovascular diseases and stroke in the US. Targeted health promotion efforts in workplaces and communities are needed to achieve these population-level changes. The present study examined the sex-specific cross-sectional associations between employment status and ideal CVH among Hispanics/Latinos, and whether these associations were modified by age (i.e., younger adults [aged 18–44] compared to middle-aged and older adults [aged 45–74]).

**Methods:**

This study included 4,797 males and 7,043 females (aged 18–74) from the Hispanic Community Health Study / Study of Latinos. Employment status was categorized as employed full-time (FT), employed part-time (PT), employed (FT or PT) and homemakers, homemakers only, and unemployed. CVH metrics, operationalized as ‘ideal’ versus ‘less than ideal,’ included health factors (i.e., blood pressure, cholesterol, and fasting glucose) and health behaviors (i.e., body mass index, smoking, physical activity [PA], and diet). A total CVH score was derived based on the seven CVH metrics, and dichotomized as ideal vs. less than ideal (score of 11–14 vs. 0–10). Survey-based generalized linear regression models with Gaussian binomial distribution were used to estimate adjusted prevalence differences (APDs) and their 95% confidence intervals (CIs) for the associations between employment status (with employed FT as referent) and ideal CVH (total score and each metric), adjusting for socio-demographic characteristics. Effect modification by age was examined.

**Results:**

Among males, compared to their employed FT counterparts, those who were employed PT had a higher prevalence of ideal CVH score (*APD* = 6.8, *95% CI* = 1.7, 11.8), ideal BMI (*APD* = 8.5, *95% CI* = 3.0, 14.0), and ideal PA (*APD* = 4.8, *95% CI* = 0.9, 8.7). Age modified the associations of employment type with ideal CVH score and ideal BMI, i.e., younger males who were employed PT had a higher prevalence of ideal CVH score and ideal BMI. Among females, employment status was not associated with ideal CVH score. Compared to females employed FT, females who were homemakers had a lower prevalence of ideal (non-) smoking (*APD* = -4.7, *95% CI* = -8.5, -1.0) and ideal PA (*APD* = -7.9, *95% CI* = -12.7, -3.0), and females who were unemployed had a lower prevalence of ideal PA (*APD* = -10.4, *95% CI* = -16.7, -4.1). Age modified the associations of employment type with ideal fasting glucose and ideal PA, i.e., middle-aged and older females who were homemakers or unemployed had a lower prevalence of ideal fasting glucose and ideal PA.

**Conclusions:**

Hispanic/Latino males who were employed PT had the most favorable CVH profiles but these associations were mostly driven by better CVH (total score and metrics) among younger males. Hispanic/Latino females who were homemakers or unemployed had lower rates of ideal CVH metrics.

## Introduction

Cardiovascular diseases (CVDs) remain a leading cause of morbidity and mortality in the United States (US) [1], exerting an enormous economic burden [2]. Consequently, there has been a growing, transformative movement to advance population-based primordial prevention in the US through the promotion and preservation of cardiovascular health (CVH) [3]. In 2010, the American Heart Association (AHA) created the 2020 Impact Goals of improving the CVH of all Americans by 20% and reducing deaths from CVDs and stroke by 20% [4]. CVH is assessed through 7 metrics (known as “Life’s Simple 7s”) which include health factors (i.e., blood pressure, cholesterol, and fasting glucose) and health behaviors (i.e., body mass index [BMI], smoking, physical activity, and diet). The presence of optimal levels of all these metrics (i.e., normal levels of blood pressure, cholesterol, and fasting glucose without drug treatment, and having normal BMI, not smoking, sufficient PA, and having a healthy diet) is defined as ideal CVH.

A growing body of prospective studies have demonstrated that the presence of ideal CVH metrics at younger age is associated with lower risk of all-cause mortality [5], lowest rate of functional disability [6], and reduced health costs [7,8] at older age. Findings from the Hispanic Community Healthy Study/Study of Latinos (HCHS/SOL) showed that the proportion of Hispanics/Latinos with ideal CVH is low (less than 1% have all 7 ideal metrics) and varies by sex with females having more favorable profiles than males [9]. Thus, targeted health promotion efforts in this segment of the US population are crucial to achieving the AHA’s 2020 Impact Goals. The Social Determinants of Health framework [10,11] posits that large improvements in population health require intervening on the broad social, economic, and physical environments where people live and work; as such, worksites may be an important setting to promote CVH among Hispanics/Latinos, the largest ethnic/racial minority in the US [12].

Previous studies have examined the associations between employment status (generally operationalized as being employed outside the home versus homemaker or as being employed versus unemployed) and CVD-related morbidity and mortality. For example, studies among middle-aged African American and non-Hispanic white women have demonstrated associations between being employed and lower prevalence and risk of hypertension [13], lower risk of coronary heart disease and ischemic stroke [14], and lower risk of all-cause mortality [15], compared to being a homemaker. Furthermore, it has been shown that the association between employment status and lower risk for incident hypertension is stronger among African American women compared to non-Hispanic white women [13], suggesting that those from lower SES may benefit more from being employed than those of higher SES. Other studies have shown that unemployed adults are more likely to smoke [16,17] and be sedentary [18] whereas females who are homemakers are less likely to exercise [19] and more likely to have excess weight [20] compared to their employed peers. In the 2008–2012 US National Health Interview Survey (NHIS), the proportion of adults (younger than 55 years) with a history of CHD/stroke was lower among those employed compared to those unemployed [21]. However, some studies have reported inconsistent results [22,23] including findings that women who are employed have a higher risk of hypertension compared to homemakers [22] and that there is no association of employment status with smoking or BMI among middle-aged women [23]. These inconsistent findings could be partially attributed to differences in assessment tools used to capture employment status and CVD risk factors across studies or in the characteristics of the study samples (e.g., age and race/ethnicity). Further, it has been shown that employment type is also associated with CVD outcomes. For instance, those employed in “lower-status” occupations such as service and blue collar workers (e.g., construction worker, factory worker, and truck driver) are more likely to report a history of CHD/stroke than those in “higher-status” occupations such as white collar workers [21].

To our knowledge, the associations between employment status and the novel concept of ideal CVH have not been comprehensively examined among adults. Previous studies on the associations between employment status and individual CVD risk factors primarily focused on non-Hispanic whites and African Americans [13–15] or had limited representation of Hispanics/Latinos (mostly Mexican Americans) in their samples [16–18,21], although Hispanics/Latinos are projected to make up nearly one third of the US population by 2050 [24]. Furthermore, the income levels of Hispanics/Latinos living in large US urban areas is lower than among non-Hispanic whites [25] but Hispanic/Latino males have one of the highest rates of labor participation in the US [26] with a significant proportion employed in blue collar occupations [27]. In contrast, Hispanic/Latina women are less likely to participate in the labor force compared to men and to non-Hispanic white women [28]. As such, the associations between employment status and CVD indicators previously documented in the literature for non-Hispanics whites and African Americans may be different among Hispanics/Latinos.

The mechanistic pathways that may explain the employment-health association are complex and remain largely unknown, although several possible mechanisms have been proposed. For example, the social causation hypothesis [29] and the well-established inverse socio-economic status (SES)-health gradient [30,31] suggest that those employed full-time (FT) may have a health advantage over the unemployed due to higher financial (e.g., stability and income) and psychological resources (e.g., increased self-esteem and perceived social support) that positively influence health. Additionally, the healthy worker effect [32,33] and social selection hypothesis [29] postulate that those with healthier profiles are more likely to enter the workforce and to remain employed than those with adverse health profiles; however, these associations could vary depending on employment type, demands, and personal resources [34]. Furthermore, women tend to have multiple roles as caregivers and homemakers in addition to being employed outside of the home, further complicating examinations of the employment-health association among women. For instance, the role-accumulation hypothesis [35] suggests that employed women have a health advantage over those who are housewives or unemployed due to higher economic independence and higher social resources. Contrastingly, the role-strain model [35] hypothesizes that FT employment among women may be detrimental to health due to the presence of psychological stressors related to maintaining multiple roles.

In this study, we examined the sex-specific cross-sectional associations between employment status and ideal CVH (i.e., total score and metrics) among Hispanics/Latinos. We hypothesized that males who are unemployed and females who are unemployed or homemakers will have a lower prevalence of ideal CVH compared to their employed FT counterparts. This study also examined whether age modifies these associations given marked differences in the prevalence of ideal CVH in younger versus middle-aged and older Hispanic/Latino adults [9,36], and the different patterns of employment status across age groups [35,37]. We hypothesized that the associations would be stronger in middle-aged and older individuals compared to younger persons. In secondary analyses limited to employed individuals, we also examined the sex-specific associations between employment type and ideal CVH. We further hypothesized that males and females in higher status occupations would have better CVH than those in lower status occupations. Such information could help to inform the development of targeted interventions for Hispanic/Latino males and females in the workplace, communities, and homes.

## Materials and methods

### Study design

The Hispanic Community Health Study/Study of Latinos has been approved by Institutional Review Boards at all study sites: the University of Illinois at Chicago (#2013–1261); University of Miami (#20131007); Albert Einstein College of Medicine (#2007–432); San Diego State University (#1586091); and University of North Carolina (#07–1003). The HCHS/SOL is a community based prospective cohort study of 16,415 non-institutionalized adults self-identifying as Hispanic/Latino and aged 18–74 years at baseline (2008–2011). Participants were selected using a stratified two-stage area probability sample of households in four field centers (Chicago, IL; Miami, FL; Bronx, NY; San Diego, CA). Persons aged 45–74 years were oversampled to enable examination of selected chronic conditions. Participants were asked to refrain from eating and smoking for 12 hours prior to the examination and to avoid physical activity the morning of the examination. Participants underwent a comprehensive examination including anthropometric assessment, blood draw, medication review, and sociodemographic and health questionnaires administered by trained, bilingual interviewers via face-to-face interviews. Additional details of study design and data collection procedures are available elsewhere [38]. The study was approved by the Institutional Review Boards at all participating study centers. Written informed consent was obtained in the language preferred by the participant and archived at each of the participating field centers.

### Analytic sample

Among the 16,415 HCHS/SOL participants, 1,545 participants who were retired and 324 participants with missing data on employment status were excluded from the current analyses. To limit reverse causality, we also excluded 2,317 participants with self-reported CVD (i.e., heart attack, angina, heart failure, peripheral arterial disease, aortic aneurysm, history of cardiovascular procedure/surgery, or stroke); chronic kidney disease; or self-reported significant physical limitations. Of the remaining 12,229 participants, 281 with missing data on CVH metrics and 108 with missing data on study covariates were excluded. These analyses are thus based on data from 11,840 participants (4,797 males and 7,043 females). Participants who were excluded from these analyses were more likely to be older, have lower educational attainment, and to be foreign-born compared to participants in the analytic sample (all *p*-values <0.001).

### Cardiovascular health

Metrics of CVH included three health factors (i.e., blood pressure, cholesterol, and fasting glucose) and four health behaviors (i.e., BMI, current smoking, physical activity, and diet). Followed by a period of 5-minutes rest, three blood pressure measurements were taken using an automatic sphygmomanometer (the OMRON HEM-907 XL); the mean of these three readings was used. Blood samples were analyzed for total serum cholesterol and fasting plasma glucose according to standardized protocols. Total cholesterol was measured in serum on a Roche Modular P Chemistry Analyzer (Roche Diagnostics Corporation) using a cholesterol oxidase enzymatic method (Roche Diagnostics, Indianapolis, IN 46250). Glucose was measured in EDTA plasma on a Roche Modular P Chemistry Analyzer (Roche Diagnostics Corporation) using a hexokinase enzymatic method (Roche Diagnostics, Indianapolis, IN 46250). Participants were asked to bring medications with them; medication names and dosages were recorded. Body weight was measured using the Tanita Body Composition Analyzer (Model TBF-300A). Height was measured to the nearest centimeter and body weight to the nearest 0.1 kg. BMI was calculated as weight in kilograms divided by height in meters squared. Smoking status and physical activity were self-reported. A modified version of the Global Physical Activity Questionnaire (GPAQ) was used to assess different activity domains (work, transport, and leisure) [39]. Dietary data were collected via two 24-hour dietary recalls administered by trained interviewers approximately 6 weeks apart [40]. Complete documentation of the examination content, laboratory procedures, and biospecimen collection and processing protocols are available at the study web site [41] (http://sites.cscc.unc.edu/hchs/protocols-and-manuals).

The AHA guidelines [3] were used to define and categorize each CVH metric and to create a total score (see Table 1). Each metric was assigned a score based on criteria for ideal (score 2), intermediate (score 1), and poor (score 0) levels. Each metric was also dichotomized as “ideal” vs. “less than ideal” (i.e., intermediate and poor categories). A total CVH score was computed by summing points for each metric. The overall CVH score was categorized into ideal (total score 11–14) versus less than ideal (total score 0–10).

**Table 1:** Definitions for the cardiovascular health (CVH) categories (i.e., less than ideal vs. ideal) according to the American Heart Association (AHA) specification.

CVH Metrics	Categories		
	IDEAL	LESS THAN IDEAL	
		Intermediate	Poor
**Health Factors**			
Blood Pressure	<120/<80 mm Hg	120–139/80-89 mm Hg or treated to control	SBP ≥140 mm Hg or DBP ≥90 mm Hg
Cholesterol	<200 mg/dL	200–239 mg/dL or treated to goal	≥240 mg/dL
Fasting Glucose	<100 mg/dL	100–125 mg/dL or treated to goal	≥126 mg/dL
**Health Behaviors**			
Body Mass Index	<25.0 kg/m^2^	25.0–29.9 kg/m^2^	≥30.0 kg/m^2^
Smoking	Never smoked or quit >12 months ago	Former smoker who quit ≤12 months ago	Current smoker
Physical Activity	≥150min/week moderate, or ≥75 min/week vigorous, or ≥150 min/week combined intensity	1–149 min/week moderate, or 1–74 min/week vigorous, or 1–149 min/week combined intensity	None
Diet*	5–4 components	3–2 components	1–0 components

SBP: systolic blood pressure; DBP: diastolic blood pressure.

*AHA diet score includes 4 criteria: ≥4.5 servings/day fruits and vegetables; ≥7oz servings/week fish; ≥3 servings/day grain; ≤4.5 servings/week sweetened beverages; and <1,500 mg/day sodium.

### Employment

Employment status was ascertained by self-reported questionnaires and categorized into 5 mutually exclusive categories: 1 = *Employed FT (>35 hours/week and not homemaker)*; 2 = *Employed part-time (PT; ≤35 hours/week and not homemaker*); 3 = *Employed (FT or PT) and homemaker*; 4 = *Homemaker*; 5 = *Unemployed (and not homemaker)*. Homemaker status was defined based a positive response to the question: “Are you a homemaker (i.e. care for family home)?” Those who were not currently employed and did not claim homemaker status were categorized as unemployed.

In secondary analyses limited to participants who were employed, employment type was categorized as: 1 = *Non-skilled worker* (i.e., ordinary laborer, construction, yard, or migrant laborer); 2 = *Service worker* (i.e., housekeeper, cook, waiter, doorkeeper, hairdresser, counter salesperson, launderer, or child care worker); 3 = *Skilled worker* (i.e., foreman, group leader, or craftsman); 4 = *Professional* (i.e., professional, technical, administrative/executive, or office staff); and 5 = *Other worker* (farmer, fisherman, hunter, army officer, police officer, soldier, policeman, driver, athlete, actor, musician, other). The *Professional* category was indicative of “higher-status” occupations, whereas the remaining categories were indicative of “lower-status” occupations.

### Covariates

Covariates used in the multivariable models included: age (continuous), education (less than high school or equivalent General Education Diploma [GED], high school or GED, greater than high school), annual household income (<$10,000, $10,001–$20,000, $20,001–$40,000, $40,001–$75,000, >$75,000, missing), Hispanic/Latino background (Cuban, Dominican, Mexican, Puerto Rican, Central American, South American, Other heritage/Mixed), current health insurance coverage (yes vs. no), years lived in the US/nativity (lived in US <10 years [and not born in the US mainland], lived in US ≥10 years [and not born in the US mainland], US-born).

### Statistical analysis

All analyses were stratified by sex because there are well-established sex-based differences in employment status [42] and the employment-health association [16,17,43]. Descriptive statistics by employment status were used to characterize the target population. The F-test (for continuous variables) and chi-square (for categorical variables) were used to detect significant differences in characteristics according to employment status; with *p*-values <0.05 deemed as statistically significant.

Survey-weighted generalized linear regression models with Gaussian binomial distribution were used to examine the associations between employment status (with employment FT as referent) and ideal CVH (including total score and individual metrics defined as binary outcomes). Adjusted prevalence differences (APDs; i.e., the difference in the prevalence of the dependent variable in the exposed group versus the unexposed/referent group) and their 95% confidence intervals (CIs) were calculated according to employment status. To improve the interpretability of our findings, the adjusted prevalence of the dependent variable for the referent group (i.e., those employed FT) is also shown in the tables.

Effect modification according to age categories (18–44 vs. ≥45 years) was assessed by adding a multiplicative interaction term to each of the final models; *P* for interaction was deemed significant at the 5% level. Finally, in secondary analyses limited to employed participants, descriptive statistics were computed by employment type. The associations between employment type and CVH with adjustment for all covariates were also examined using survey-weighted generalized linear regression models with Gaussian binomial distribution. Effect modification by age was assessed by adding multiplicative interaction terms to the final model.

Data management was performed using SAS 9.4 software (SAS Institute, Cary, NC) and all statistical analyses were performed using Stata Statistical Software Release 15 (Stata Corp LP, College Station, TX). Models were adjusted for socio-demographic characteristics: age (continuous), education, annual household income, Hispanic/Latino background, health insurance status, and years lived in the US/nativity. All analyses were weighted to account for the complex sampling design and non-response bias.

## Results

### Descriptive characteristics by employment status

Table 2 presents characteristics of the target population for males according to employment status. Among males, 43.5% were employed FT and 4.6% were homemakers. Males who were employed PT or unemployed were, on average, younger than others (*p*<0.001). Males who were employed FT were more likely to report higher education (*p*<0.001) and higher annual household income levels (*p*<0.001) compared to other groups. Table 2 also presents the unadjusted prevalence of ideal CVH (total score and metrics) by employment status. Among males, those who were employed PT had the highest unadjusted prevalence of ideal CVH score (27%; *p*<0.001), ideal blood pressure 48.9%; (*p*<0.001), ideal BMI (29.9%; *p*<0.001), and ideal physical activity (85.2%; *p*<0.001). Males who were unemployed had a higher unadjusted prevalence of ideal cholesterol (60.2%; *p*<0.001) and males who were employed FT had a higher prevalence of ideal (non-) smoking (76.2%; *p*<0.001). Males who were employed PT or unemployed had the highest prevalence of ideal diet (1.4% each; *p*<0.05).

**Table 2:** Descriptive characteristics by employment status among males (*n* = 4,797), HCHS/SOL (2008–2011).

	Employed Full-Time (*n* = 2,215)	Employed Part-Time (*n* = 668)	Employed and Homemaker (*n* = 416)	Homemaker (*n* = 242)	Unemployed (*n* = 1,346)
	Mean or Proportion (95% Confidence Interval)^a^				
Proportion	**43.5 (41.3, 45.7)**	**15.0 (13.6, 16.4)**	**7.5 (6.6, 8.5)**	**4.6 (3.8, 5.4)**	**29.4 (27.4, 31.5)**
**Age**, Mean***	38.4 (37.6, 39.1)	35.2 (33.9, 36.6)	39.9 (38.5, 41.3)	39.9 (37.7, 42.1)	35.6 (34.4, 36.7)
**Education**, %***					
<High School	26.9 (24.2, 29.7)	26.8 (22.7, 31.3)	30.8 (25.4, 36.8)	36.9 (28.9, 45.6)	31.7 (28.5, 35.1)
High School	29.0 (26.2, 31.9)	34.1 (29.5, 39.2)	34.4 (28.7, 40.5)	31.9 (24.4, 40.5)	36.2 (32.9, 39.8)
>High School	44.1 (40.6, 47.7)	39.1 (34.0, 44.4)	34.8 (29.0, 41.0)	31.2 (23.7, 39.9)	32.0 (28.7, 35.6)
**Annual Household Income**, %***					
<$10,000	3.0 (2.1, 4.1)	9.0 (6.6, 12.1)	9.2 (5.7, 14.5)	16.6 (11.6, 23.2)	16.1 (13.5, 19.0)
$10,001–$20,000	20.9 (18.2, 24.0)	31.4 (26.9, 36.3)	29.0 (23.5, 35.2)	29.0 (22.1, 37.1)	31.5 (28.4, 34.8)
$20,001–$40,000	39.2 (36.1, 42.4)	32.4 (27.7, 37.5)	35.1 (29.4, 41.3)	31.8 (24.2, 40.5)	24.0 (21.3, 27.0)
$40,001–$75,000	22.4 (20.0, 25.1)	14.5 (11.0, 19.0)	15.1 (11.1, 20.0)	7.7 (4.1, 14.1)	10.9 (8.7, 13.5)
>$75,000	11.3 (8.8, 14.5)	5.6 (3.0, 10.1)	6.2 (3.5, 10.5)	4.1 (1.2, 13.0)	3.9 (2.2, 6.9)
Missing	3.1 (2.3, 4.3)	7.1 (4.8, 10.3)	5.5 (2.9, 10.1)	10.7 (5.8, 19.0)	13.6 (11.1, 16.5)
**Hispanic/Latino Background**, %***					
Cuban	18.6 (15.0, 22.7)	15.0 (11.0, 20.2)	22.4 (16.9, 29.1)	15.6 (10.5, 22.7)	28.5 (23.4, 34.1)
Dominican	5.9 (4.5, 7.7)	5.9 (3.9, 8.7)	11.3 (8.0, 15.7)	13.8 (8.4, 21.9)	8.0 (6.1, 10.6)
Mexican	46.7 (42.1, 51.3)	43.2 (37.1, 49.5)	37.9 (31.6, 44.5)	25.6 (18.8, 33.8)	31.8 (27.0, 37.0)
Puerto Rican	12.7 (10.5, 15.4)	13.6 (10.0, 18.3)	10.8 (7.6, 15.2)	25.5 (18.3, 34.2)	14.8 (12.3, 17.8)
Central American	7.1 (5.8, 8.8)	9.7 (7.1, 13.1)	7.3 (4.8, 11.0)	6.8 (4.0, 11.3)	9.1 (7.1, 11.6)
South American	5.1 (4.1, 6.4)	6.5 (4.6, 9.0)	6.5 (4.3, 9.6)	4.8 (2.6, 8.6)	3.8 (2.8, 5.1)
Other/Mixed	3.8 (2.7, 5.4)	6.1 (3.8, 9.8)	3.8 (1.6, 8.8)	7.9 (3.8, 15.9)	4.0 (2.9, 5.5)
**Current Health Insurance**, %*					
Yes	43.5 (40.1, 46.9)	37.3 (31.9, 43.0)	34.1 (28.2, 40.6)	45.9 (37.4, 54.6)	40.6 (36.7, 44.5)
No	56.5 (53.1, 59.9)	62.7 (57.0, 68.1)	65.9 (59.4, 71.8)	54.1 (45.4, 62.6)	59.4 (55.5, 63.3)
**Years in the US/Nativity**, %***					
<10 Years	30.7 (27.6, 34.0)	31.1 (26.4, 36.1)	28.5 (23.2, 34.4)	19.1 (13.7, 26.0)	29.3 (25.5, 33.5)
≥10 Years	48.1 (45.1, 51.1)	42.4 (37.5, 47.3)	51.7 (45.4, 57.9)	48.5 (39.8, 57.3)	36.3 (32.6, 40.1)
US-Born	21.2 (18.6, 24.1)	26.6 (22.1, 31.6)	19.9 (14.5, 26.5)	32.4 (24.0, 42.0)	34.4 (30.0, 39.1)
**Ideal Cardiovascular Health (CVH)**					
Total Score, %***	19.0 (16.9, 21.3)	27.0 (22.3, 32.3)	14.5 (10.9, 19.0)	13.7 (8.9, 20.7)	18.7 (16.0, 21.7)
**Ideal CVH Metrics**					
Blood Pressure, %	42.9 (39.9, 46.0)	48.9 (43.4, 54.4)	40.3 (34.5, 46.4)	43.9 (35.2, 53.0)	44.3 (40.6, 48.0)
Cholesterol, %***	50.1 (47.0, 53.2)	57.5 (52.3, 62.6)	56.6 (50.4, 62.6)	55.6 (47.1, 63.8)	60.2 (56.4, 63.8)
Fasting Glucose, %	64.2 (61.4, 67.0)	70.2 (65.3, 74.6)	64.4 (58.4, 70.0)	61.9 (53.4, 69.7)	65.7 (62.1, 69.1)
BMI, %***	19.0 (16.8, 21.3)	29.9 (25.1, 35.2)	20.7 (16.0, 26.2)	20.4 (14.6, 27.7)	26.9 (24.0, 30.0)
(non-) Smoking, %***	76.2 (73.5, 78.7)	71.1 (66.4, 75.3)	71.7 (65.4, 77.2)	64.9 (56.1, 72.8)	62.6 (59.1, 65.9)
Physical Activity, %***	78.5 (75.9, 80.9)	85.2 (81.6, 88.2)	81.4 (76.0, 85.9)	78.4 (71.6, 83.9)	72.9 (69.5, 76.0)
Diet, %*	1.1 (0.7, 1.8)	1.4 (0.6, 2.9)	1.3 (0.6, 2.9)	0.0 (0.0, 0.0)	1.4 (0.6, 3.0)

^a^ Means and proportions are weighted (except sample sizes)

*p<0.05

**p<0.01

***p<0.001

Among females, homemakers comprised the largest group (37.8%) (Table 3). On average, females who were unemployed were younger than others (*p*<0.001). Females who were employed FT had higher education (*p*<0.001) and annual household income levels (*p*<0.001), and were more likely to have health insurance than other groups (*p*<0.001). Among females, those employed PT had the highest unadjusted prevalence of ideal CVH score (34.8%; *p*<0.001), ideal cholesterol (74.0%; *p*<0.001), and ideal physical activity (68.7%; *p*<0.001); females who were unemployed had the highest prevalence of ideal blood pressure (79.0%; *p*<0.001) and ideal BMI (38.3%; *p*<0.001). Females who were employed/homemakers had the highest prevalence of ideal (non-) smoking (87.2%; *p*<0.001) and ideal diet (2.3%; *p*<0.05) (Table 3).

**Table 3:** Descriptive characteristics by employment status among females (*n* = 7,043), HCHS/SOL (2008–2011).

	Employed Full-Time (*n* = 1,047)	Employed Part-Time (*n* = 580)	Employed and Homemaker (*n* = 2,091)	Homemaker (*n* = 2,677)	Unemployed (*n* = 648)
	Mean or Proportion (95% Confidence Interval)^a^				
Proportion	**14.4 (13.2, 15.6)**	**10.1 (8.9, 11.4)**	**24.5 (22.9, 26.1)**	**37.8 (36.0, 39.7)**	**13.2 (11.9, 14.6)**
**Age**, Mean***	38.0 (36.9, 39.0)	32.0 (30.7, 33.2)	42.5 (41.8, 43.3)	41.5 (40.7, 42.3)	28.5 (27.5, 29.5)
**Education**, %***					
<High School	12.7 (10.6, 15.3)	21.1 (15.4, 28.1)	34.6 (31.4, 37.9)	39.6 (36.7, 42.5)	21.6 (18.0, 25.8)
High School	23.2 (19.7, 27.2)	28.3 (23.4, 33.7)	26.2 (23.7, 29.0)	29.1 (26.3, 32.0)	31.1 (27.0, 35.6)
>High School	64.1 (59.7, 68.2)	50.7 (44.8, 56.5)	39.2 (36.0, 42.4)	31.3 (27.9, 35.0)	47.2 (42.2, 52.3)
**Annual Household Income**, %***					
<$10,000	5.0 (3.6, 6.9)	7.6 (5.4, 10.5)	12.9 (10.7, 15.3)	18.2 (15.7, 21.0)	14.2 (11.4, 17.6)
$10,001–$20,000	19.4 (16.5, 22.7)	33.0 (27.9, 38.6)	34.7 (31.8, 37.7)	33.6 (30.9, 36.5)	25.1 (20.9, 29.9)
$20,001–$40,000	37.7 (33.2, 42.3)	32.8 (27.1, 39.0)	32.8 (30.1, 35.5)	25.8 (23.1, 28.7)	26.7 (22.4, 31.6)
$40,001–$75,000	22.3 (18.5, 26.7)	16.4 (11.6, 22.8)	11.3 (9.5, 13.5)	8.0 (6.5, 9.7)	10.2 (7.3, 14.1)
>$75,000	10.5 (8.1, 13.5)	2.2 (1.1, 4.3)	3.1 (2.1, 4.6)	2.8 (1.4, 5.7)	4.0 (2.5, 6.3)
Missing	5.1 (3.5, 7.5)	8.0 (5.6, 11.1)	5.2 (3.9, 6.9)	11.6 (10.0, 13.4)	19.7 (16.0, 24.1)
**Hispanic/Latino Background**, %***					
Cuban	19.3 (15.4, 23.9)	14.5 (10.3, 19.9)	11.0 (8.4, 14.1)	18.2 (14.6, 22.5)	26.1 (20.5, 32.5)
Dominican	6.4 (4.4, 9.1)	9.3 (6.4, 13.3)	15.2 (12.6, 18.3)	9.8 (7.6, 12.6)	10.6 (7.6, 14.6)
Mexican	41.2 (36.1, 46.5)	47.3 (40.4, 54.2)	43.3 (39.0, 47.7)	47.0 (42.5, 51.6)	32.3 (26.8, 38.3)
Puerto Rican	14.0 (11.4, 17.1)	8.9 (6.1, 12.7)	8.5 (7.1, 10.2)	12.3 (10.1, 14.8)	14.1 (10.9, 18.0)
Central American	8.7 (6.7, 11.3)	7.4 (5.4, 10.1)	11.7 (9.4, 14.4)	6.4 (5.2, 7.8)	5.5 (3.9, 7.7)
South American	5.2 (3.8, 7.1)	6.4 (4.5, 8.9)	6.8 (5.4, 8.5)	4.2 (3.3, 5.4)	3.7 (2.2, 6.3)
Other/Mixed	5.2 (3.5, 7.8)	6.4 (2.8, 13.8)	3.6 (2.3, 5.5)	2.1 (1.5, 3.0)	7.8 (5.3, 11.3)
**Current Health Insurance**, %***					
Yes	57.7 (53.1, 62.1)	44.6 (37.5, 51.9)	44.7 (41.2, 48.2)	46.3 (43.2, 49.4)	49.3 (44.5, 54.1)
No	42.3 (37.9, 46.9)	55.4 (48.1, 62.5)	55.3 (51.8, 58.8)	53.7 (50.6, 56.8)	50.7 (45.9, 55.5)
**Years in the US/Nativity**, %***					
<10 Years	28.0 (24.0, 32.3)	23.2 (18.1, 29.3)	31.4 (28.1, 34.9)	33.1 (29.9, 36.5)	28.8 (23.6, 34.7)
≥10 Years	43.8 (38.6, 47.1)	38.7 (32.0, 45.8)	57.8 (54.4, 61.1)	51.1 (48.0, 54.3)	24.9 (20.7, 29.8)
US-Born	29.2 (25.2, 33.6)	38.1 (31.9, 44.6)	10.8 (9.0, 13.0)	15.7 (13.1, 18.7)	46.2 (40.1, 52.5)
**Ideal Cardiovascular Health (CVH)**					
Total Score, %***	31.5 (27.3, 36.1)	34.8 (29.4, 40.7)	24.5 (21.8, 27.4)	21.5 (18.5, 24.7)	33.4 (28.9, 38.2)
**Ideal CVH Metrics**					
Blood Pressure, %***	67.2 (62.9, 71.1)	75.6 (70.7, 79.9)	60.8 (57.7, 63.8)	59.1 (55.7, 62.5)	79.0 (75.1, 82.4)
Cholesterol, %***	57.1 (52.6, 61.4)	74.0 (68.7, 78.7)	53.0 (49.9, 56.2)	54.2 (51.2, 57.1)	72.8 (68.1, 77.1)
Fasting Glucose, %	81.1 (77.0, 84.7)	88.3 (84.8, 91.0)	73.9 (71.2, 76.4)	72.9 (70.1, 75.4)	84.7 (81.3, 87.5)
BMI, %***	29.1 (24.7, 34.0)	31.1 (25.8, 36.9)	21.9 (19.5, 24.6)	20.5 (18.1, 23.1)	38.3 (33.0, 44.0)
(non-) Smoking, %***	85.9 (83.0, 88.3)	80.6 (73.3, 86.3)	87.2 (85.2, 89.0)	80.3 (77.5, 82.7)	76.9 (72.2, 81.0)
Physical Activity, %***	66.3 (62.2, 70.1)	68.7 (63.8, 73.3)	64.2 (61.2, 67.2)	55.2 (52.4, 57.9)	57.7 (52.8, 62.5)
Diet, %*	2.0 (1.2, 3.2)	1.1 (0.3, 4.2)	2.3 (1.5, 3.4)	2.2 (1.6, 3.1)	0.3 (0.1, 0.9)

^a^ Means and proportions are weighted (n’s are unweighted)

*p<0.05

**p<0.01

***p<0.001

### Associations between employment status and ideal CVH

Table 4 shows estimates for the sex-specific adjusted associations between employment status (with employed FT status serving as the referent category) and ideal CVH score and metrics (all ages). Males who were employed PT (vs. FT) had a 7% higher prevalence of ideal CVH score (*APD* = 6.8, *95% CI* = 1.7, 11.8). In analyses on individual CVH metrics, males who were employed PT (vs. FT) had a 9% and 5% higher prevalence of ideal BMI and ideal physical activity (*APD* = 8.5, *95% CI* = 3.0, 14.0; and *APD* = 4.8, *95% CI* = 0.9, 8.7, respectively), and males who were employed and homemakers (vs. employed FT) had a 8% higher prevalence of ideal cholesterol (*APD* = 8.0, *95% CI* = 1.5, 14.5). Males who were unemployed (vs. employed FT) had a 4% higher prevalence of ideal BMI (*APD* = 4.4, *95% CI* = 0.6, 8.3) but also had a 9% and 6% lower prevalence of ideal (non-) smoking and ideal physical activity (*APD* = -9.2, *95% CI* = -14.1, -4.4; and *APD* = -5.7, *95% CI* = -9.5, -1.9, respectively).

**Table 4:** Associations between employment status and ideal cardiovascular health (CVH) by sex and age groups: Adjusted^†^ prevalence differences (APD) with 95% confidence interval, HCHS/SOL (2008–2011).

	ALL AGES		MALES		FEMALES	
	Males (*n* = 4,797)	Females (*n* = 7,043)	Ages 18–44 (*n* = 2,465)	Ages 45–74 (*n* = 2,332)	Ages 18–44 (*n* = 3,234)	Ages 45–74 (*n* = 3,809)
APD (95% CI)						
**Ideal CVH**						
*Employed FT (ref)*	*18*.*5 (16*.*3*, *20*.*7)*	*29*.*3 (25*.*4*, *33*.*1)*	*22*.*2 (19*.*3*, *25*.*1)*	*11*.*7 (9*.*1*, *14*.*3)*	*38*.*0 (32*.*6*, *43*.*3)*	*12*.*4 (8*.*5*, *16*.*2)*
Employed PT	**6.8 (1.7, 11.8)**	-1.3 (-8.1, 5.5)	**9.2 (2.8, 15.7)**	-3.8 (-7.9, 0.3)	-2.9 (-11.4, 5.7)	1.9 (-5.4, 9.1)
Employed/Homemaker	-1.6 (-6.2, 2.9)	-1.8 (-6.8, 3.1)	-0.9 (-7.7, 5.8)	-3.8 (-8.0, 0.4)	-1.3 (-8.6, 6.0)	-1.6 (-6.3, 3.1)
Homemaker	-0.7 (-6.5, 5.4)	-4.2 (-9.1, 0.6)	2.7 (-6.3, 11.8)	**-7.5 (-12.0, -3.0)**	-3.8 (-10.7, 3.0)	**-5.1 (-9.3, -0.8)**
Unemployed	0.5 (-3.0, 4.1)	-3.7 (-9.7, 2.3)	1.6 (-3.1, 6.2)	**-6.7 (-10.1, -3.2)**	-5.6 (-13.5, 2.3)	-1.0 (-6.8, 4.9)
				*P*_*interaction*_ = **0.002**		*P*_*interaction*_ = 0.948
**Ideal Blood Pressure**						
*Employed FT (ref)*	*43*.*2 (4020*, *46*.*3)*	*64*.*5 (61*.*1*, *68*.*0)*	*49*.*4 (45*.*3*, *53*.*6)*	*27*.*3 (23*.*9*, *30*.*7)*	*79*.*4 (74*.*7*, *84*.*1)*	*34*.*0 (28*.*5*, *39*.*4)*
Employed PT	3.2 (-3.0, 9.4)	-1.4 (-7.0, 4.1)	2.9 (-4.7, 10.6)	7.5 (-0.8, 15.8)	-1.0 (-7.8, 5.9)	0.9 (-8.3, 10.0)
Employed/Homemaker	-0.0 (-6.6, 6.6)	2.7 (-1.6, 7.1)	2.1 (-7.7, 11.9)	-3.6 (-11.0, 3.8)	4.3 (-1.9, 10.5)	0.6 (-5.9, 7.0)
Homemaker	4.5 (-4.3, 13.4)	0.4 (-4.3, 5.0)	10.6 (-0.8, 22.0)	-6.1 (-19.1, 6.8)	0.5 (-5.5, 6.4)	0.3 (-6.5, 7.0)
Unemployed	0.5 (-4.5, 5.4)	-1.6 (-6.5, 3.3)	2.5 (-3.9, 9.0)	-1.3 (-7.4, 4.9)	-1.4 (-7.6, 4.7)	7.7 (-2.1, 17.4)
				*P*_*interaction*_ = 0.365		*P*_*interaction*_ = 0.743
**Ideal Cholesterol**						
*Employed FT (ref)*	*52*.*5 (49*.*4*, *55*.*6)*	*56*.*9 (52*.*8*, *61*.*0)*	*60*.*6 (57*.*0*, *64*.*3)*	*37*.*6 (33*.*0*, *42*.*2)*	*71*.*5 (65*.*7*, *77*.*2)*	*28*.*3 (23*.*5*, *33*.*2)*
Employed PT	2.7 (-3.1, 8.4)	6.0 (-0.1, 12.1)	1.1 (-5.7, 7.9)	-1.6 (-10.6, -7.4)	7.5 (-0.2, 15.2)	1.5 (-7.4, 10.4)
Employed/Homemaker	**8.0 (1.5, 14.5)**	3.1 (-1.8, 8.1)	6.2 (-1.6, 13.9)	9.2 (-1.4, 19.7)	1.1 (-5.8, 8.0)	4.7 (-1.6, 11.0)
Homemaker	4.5 (-4.3, 13.2)	2.2 (-2.6, 6.9)	1.4 (-8.5, 11.4)	7.3 (-6.6, 21.3)	3.5 (-3.1, 10.2)	-1.2 (-7.5, 5.1)
Unemployed	4.1 (-0.4, 8.6)	-2.1 (-8.3, 4.0)	1.8 (-3.7, 7.2)	0.5 (-6.4, 7.4)	-2.0 (-10.1, 5.9)	1.4 (-7.0, 9.8)
				*P*_*interaction*_ = 0.288		*P*_*interaction*_ = **0.001**
**Ideal Fasting Glucose**						
*Employed FT (ref)*	*66*.*2 (63*.*4*, *69*.*0)*	*78*.*3 (74*.*3*, *82*.*3)*	*73*.*9 (70*.*4*, *77*.*4)*	*49*.*4 (45*.*4*, *53*.*4)*	*85*.*1 (79*.*9*, *90*.*3)*	*65*.*8 (61*.*0*, *70*.*5)*
Employed PT	1.6 (-3.7, 6.8)	3.1 (-2.2, 8.3)	2.1 (-4.0, 8.2)	-3.1 (-11.8, 5.5)	4.9 (-1.6, 11.3)	1.3 (-6.8, 9.3)
Employed/Homemaker	1.4 (-4.6, 7.5)	-0.0 (-4.4, 4.4)	-0.4 (-8.2, 7.5)	3.3 (-6.5, 13.0)	0.6 (-5.3, 6.5)	-4.0 (-9.9, 1.9)
Homemaker	-1.7 (-9.3, 6.0)	-1.4 (-5.9, 3.0)	-0.5 (-10.1, 9.0)	-5.0 (-17.9, 8.0)	1.6 (-4.0, 7.3)	**-9.5 (-16.1–2.9)**
Unemployed	-3.4 (-8.1, 1.4)	-4.8 (-9.8, 0.1)	-3.8 (-9.9, 2.2)	-4.6 (-11.5, 2.3)	-0.9 (-7.2, 5.4)	**-11.1 (-21.1, -1.1)**
				*P*_*interaction*_ = 0.419		*P*_*interaction*_ = **0.002**
**Ideal BMI**						
*Employed FT (ref)*	*20*.*3 (17*.*9*, *22*.*8)*	*26*.*8 (22*.*7*, *30*.*8)*	*22*.*1 (18*.*8*, *25*.*4)*	*19*.*3 (15*.*9*, *22*.*7)*	*30*.*9 (25*.*5*, *36*.*3)*	*19*.*2 (14*.*6*, *23*.*9)*
Employed PT	**8.5 (3.0, 14.0)**	0.1 (-6.6, 6.8)	**9.3 (2.3, 16.2)**	-0.1 (-7.7, 7.5)	-1.6 (-9.7, 6.5)	0.4 (-8.4, 7.5)
Employed/Homemaker	2.4 (-3.0, 7.8)	-2.0 (-7.1, 3.0)	3.1 (-4.2, 10.3)	-0.8 (-7.9, 6.2)	-1.7 (-8.5, 5.0)	0.1 (-5.7, 5.8)
Homemaker	1.1 (-5.4, 7.6)	-3.8 (-8.3, 0.7)	5.1 (-4.5, 14.7)	-6.4 (-13.5, 0.7)	-2.1 (-8.4, 4.1)	-5.4 (-10.8, -0.0)
Unemployed	**4.4 (0.6, 8.3)**	4.9 (-1.6, 11.4)	4.8 (-0.2, 9.8)	-3.7 (-8.8, 1.3)	0.5 (-7.5, 8.5)	5.3 (-2.5, 13.0)
				*P*_*interaction*_ = **0.038**		*P*_*interaction*_ = 0.174
**Ideal (non-) Smoking**						
*Employed FT (ref)*	*74*.*3(71*.*5*, *77*.*1)*	*85*.*1 (82*.*5*, *87*.*8)*	*74*.*6(71*.*0*, *78*.*2)*	*74*.*9 (71*.*4*, *78*.*5)*	*86*.*3 (82*.*6*, *90*.*0)*	*82*.*5 (79*.*0*, *86*.*1)*
Employed PT	-2.4 (-7.9, 2.5)	-3.9 (-10.7, 2.9)	-2.9 (-9.4, 3.5)	-6.0 (-15.0, 3.1)	-5.4 (-13.8, 2.9)	-0.0 (-7.3, 7.3)
Employed/Homemaker	-3.2 (-9.1, 2.6)	-0.1 (-3.4, 3.1)	-4.3 (-12.7, 4.1)	-0.7 (-9.6, 8.1)	0.5 (-4.4, 5.5)	1.0 (-3.3, 5.4)
Homemaker	-7.7 (-16.5, 1.1)	**-4.7 (-8.5, -1.0)**	-9.6 (-21.6, 2.4)	-4.7 (-15.8, 6.3)	**-6.1 (-11.1, -1.0)**	-1.7 (-6.2, 2.8)
Unemployed	**-9.2 (-14.1, -4.4)**	-4.3 (-9.5, 0.9)	**-10.4 (-16.4, -4.3)**	**-9.5 (-16.4, -2.6)**	-4.7 (-11.1, 1.7)	**-11.9 (-20.4, -3.3)**
				*P*_*interaction*_ = 0.987		*P*_*interaction*_ = 0.073
**Ideal Physical Activity**						
*Employed FT (ref)*	*78*.*7 (76*.*3 81*.*1)*	*65*.*1 (61*.*1*, *69*.*1)*	*82*.*7 (79*.*8*, *85*.*5)*	*69*.*6 (65*.*4*, *73*.*8)*	*67*.*5 (62*.*0*, *73*.*1)*	*61*.*0 (55*.*4*, *66*.*6)*
Employed PT	**4.8 (0.9, 8.7)**	-0.6 (-6.4, 5.2)	4.0 (-0.8, 8.7)	6.9 (-0.4, 14.2)	-2.5 (-9.8, 4.8)	5.1 (-5.0, 15.2)
Employed/Homemaker	4.4 (-1.0, 9.7)	0.1 (-4.8, 5.0)	5.1 (-1.8, 12.0)	3.1 (-5.4, 11.7)	-0.1 (-7.3, 7.0)	0.5 (-6.1, 7.0)
Homemaker	1.1 (-5.3, 7.4)	**-7.9 (-12.7, -3.0)**	0.0 (-8.0, 8.1)	2.9 (-7.5, 13.3)	-5.1 (-12.2, 2.0)	**-12.8 (-20.1, -5.4)**
Unemployed	**-5.7 (-9.5, -1.9)**	**-10.4 (-16.7, -4.1)**	-4.5 (-9.3, 0.3)	**-9.7 (-16.6, -2.8)**	**-10.6 (-18.9, -2.4)**	**-17.3 (-27.1, -7.5)**
				*P*_*interaction*_ = 0.498		*P*_*interaction*_ = **0.016**
**Ideal Diet**						
*Employed FT (ref)*	*0*.*9 (0*.*3*, *1*.*4)*	*2*.*0 (0*.*9*, *3*.*1)*	*0*.*6 (-0*.*1*, *1*.*4)*	*1*.*4 (0*.*7*, *2*.*0)*	*1*.*6 (0*.*3*, *2*.*9)*	*3*.*0 (1*.*0*, *5*.*0)*
Employed PT	0.5 (-0.6, 1.7)	-0.7 (-2.5, 1.2)	0.7 (-0.9, 2.3)	0.1 (-1.3, 1.5)	0.3 (-2.5, 2.0)	-1.6 (-4.4, 1.2)
Employed/Homemaker	0.4 (-0.8, 1.6)	-0.0 (-1.5, 1.4)	-0.4 (-1.4, 0.6)	1.6 (-0.9, 4.2)	0.2 (-1.4, 1.9)	-0.9 (-3.4, 1.6)
Homemaker	-0.6 (-1.4, 0.3)	0.0 (-1.5, 1.5)	-0.2 (-1.4, 1.0)	**-1.4 (-2.3, -0.5)**	0.1 (-1.7, 1.9)	-0.4 (-2.8, 1.9)
Unemployed	-0.9 (-0.9, 2.7)	-0.9 (-2.2, 0.4)	1.3 (-1.2, 3.9)	-0.2 (-1.2, 0.8)	-0.5 (-2.1, 1.1)	-2.2 (-4.4, -0.0)
			*P*_*interaction*_ = 0.363		*P*_*interaction*_ = 0.962	

Results are weighted (*n*’s are unweighted). ^†^Adjusted for age (continuous), education, annual household income, Hispanic/Latino background, health insurance status, and years lived in the US/nativity

Among females, no association between employment status and ideal CVH score was observed. In analyses on individual CVH metrics, females who were homemakers (vs. employed FT) had a 5% and 8% lower prevalence of ideal (non-) smoking and ideal physical activity (*APD* = -4.7, *95% CI* = -8.5, -1.0; and *APD* = -7.9, *95% CI* = -12.7, -3.0, respectively). Females who were unemployed had a 10% lower prevalence of ideal physical activity (*APD* = -10.4, *95% CI* = -16.7, -4.1).

Among males, age modified the association of employment status with ideal CVH score (*P*_*interaction*_ = 0.002) and ideal BMI (*P*_*interaction*_ = 0.038). Males aged 18–44 years who were employed PT (vs. FT) had a 9% higher prevalence of ideal CVH score (*APD* = 9.2, *95% CI* = 2.8, 15.7) and a 9% higher prevalence of ideal BMI (*APD* = 9.3, *95% CI*: 2.3, 16.2). Males aged 45–74 years who were homemakers and those who were unemployed (vs. employed FT) had an 8% and 7% lower prevalence of ideal CVH score, respectively (*APD* = -7.5, *95% CI* = 12.0, -3.0; and *APD* = -6.7, *95% CI* = -10.1, -3.2, respectively) (Table 4).

Among females, age was an effect modifier in the associations of employment status with ideal cholesterol (*P*_*interaction*_ = 0.001), ideal fasting glucose (*P*_*interaction*_ = 0.002), and ideal physical activity (*P*_*interaction*_ = 0.016). In age-stratified analyses, however, no association was seen between employment status and ideal cholesterol among females. Among females aged 45–74 years, those who were homemakers and those who were unemployed (vs. employed FT) each had a 10% and 11% lower prevalence of ideal fasting glucose (*APD* = -9.5, *95% CI* = -16.1, -2.9; and *APD* = -11.1, *95% CI* = -21.1, -1.1, respectively) and those who were homemakers (vs. employed FT) had a 13% lower prevalence of ideal physical activity (*APD* = -12.8, *95% CI*: -20.1, -5.4). Among females ages 18–44 and 45–74 years, those who were unemployed (vs. employed FT) had a 11% and 17% lower prevalence of ideal physical activity (*APD* = -10.6, *95% CI*: -18.9, -2.4; and *APD* = -17.3, *95% CI*: -27.1, -7.5, respectively) (Table 4).

### Results of secondary analyses

Table 5 shows descriptive characteristics for employed males according to employment type. Among males, the predominant employment type was non-skilled worker (30.4%), while professional worker was the least common (11.3%). Compared to other employment types, males who were professional workers had higher education (*p*<0.001) and annual household income (*p*<0.001) levels. Professional workers had the highest unadjusted prevalence of ideal (non-) smoking (83.1%; *p*<0.01) and ideal diet (1.5%; *p*<0.01). There were no significant differences in the unadjusted prevalence of ideal CVH, ideal blood pressure, ideal cholesterol, ideal fasting glucose, ideal BMI, or ideal physical activity across employment type categories in men (Table 5).

**Table 5:** Descriptive characteristics by employment status among employed males (*n* = 3,209), HCHS/SOL (2008–2011).

	Non-Skilled Worker (*n* = 1,062)	Service Worker (*n* = 401)	Skilled Worker (*n* = 853)	Professional Worker (*n* = 313)	Other Worker (*n* = 580)
	Mean or Proportion (95% Confidence Interval)^a^				
Proportion	**30.4 (27.9, 32.9)**	**13.2 (11.6, 14.9)**	**25.8 (23.5, 28.2)**	**11.3 (9.1, 13.9)**	**19.4 (17.4, 21.6)**
**Age**, Mean	36.9 (35.9, 37.9)	38.3 (36.5, 40.0)	38.5 (37.3, 39.7)	39.1 (37.7, 40.6)	37.4 (36.0, 38.8)
**Education**, %***					
<High School	37.6 (33.5, 41.9)	19.0 (14.6, 24.4)	29.0 (24.5, 33.9)	5.8 (3.5, 9.5)	27.0 (22.3, 32.3)
High School	36.3 (32.0, 40.8)	34.2 (28.4, 40.6)	26.9 (23.2, 30.9)	19.3 (13.1, 27.4)	31.7 (26.4, 37.6)
>High School	26.1 (22.4, 30.3)	46.8 (40.3, 53.4)	44.2 (39.4, 49.1)	74.9 (66.3, 81.9)	41.3 (35.5, 47.3)
**Annual Household Income**, %***					
<$10,000	6.2 (4.4, 8.6)	7.0 (4.2, 11.3)	4.6 (3.1, 6.7)	1.5 (0.5, 4.4)	4.7 (3.1, 7.2)
$10,001–$20,000	32.1 (27.9, 36.7)	24.3 (19.5, 29.7)	23.0 (18.9, 27.7)	10.2 (6.2, 16.6)	21.6 (17.1, 26.8)
$20,001–$40,000	38.5 (34.4, 42.7)	38.4 (32.4, 44.9)	36.6 (31.8, 41.8)	21.1 (16.0, 27.4)	44.4 (38.9, 50.1)
$40,001–$75,000	13.4 (10.9, 16.4)	20.2 (14.6, 27.1)	24.0 (19.9, 28.7)	32.1 (26.1, 38.7)	16.9 (13.1, 21.4)
>$75,000	5.0 (3.0, 8.3)	4.3 (2.3, 8.0)	8.2 (6.0, 11.2)	32.5 (24.7, 41.5)	7.9 (4.9, 12.6)
Missing	4.8 (3.3, 7.1)	5.9 (3.5, 9.7)	3.5 (2.2, 5.7)	2.5 (0.7, 8.4)	4.5 (2.9, 7.0)
**Hispanic/Latino Background**, %***					
Cuban	10.7 (7.8, 14.4)	35.4 (28.8, 42.8)	12.7 (9.5, 16.7)	15.4 (10.6, 21.8)	27.3 (21.0, 34.6)
Dominican	6.5 (4.7, 8.9)	1.6 (0.8, 3.2)	8.8 (6.6, 11.7)	4.9 (2.5, 9.4)	7.7 (5.3, 11.1)
Mexican	54.7 (49.5, 59.9)	35.7 (28.3, 43.8)	47.7 (41.9, 53.5)	38.2 (28.8, 48.5)	35.9 (29.7, 42.6)
Puerto Rican	11.2 (8.4, 14.6)	5.8 (3.4, 9.8)	15.8 (12.3, 20.2)	20.8 (15.5, 27.5)	11.0 (6.8, 17.4)
Central American	10.5 (8.0, 13.7)	9.2 (6.4, 13.0)	5.7 (4.1, 7.9)	49.9 (2.9, 8.1)	6.9 (4.7, 10.0)
South American	4.4 (3.1, 6.1)	8.1 (5.6, 11.5)	4.7 (3.6, 6.3)	5.3 (3.3, 8.5)	7.1 (5.0, 9.9)
Other/Mixed	2.2 (1.2, 3.7)	4.2 (2.2, 7.9)	4.5 (2.6, 7.7)	10.4 (6.3, 16.8)	4.1 (2.4, 7.1)
**Current Health Insurance**, %***					
Yes	35.6 (31.4, 39.9)	32.4 (26.3, 39.1)	44.5 (39.6, 49.6)	60.5 (52.5, 68.0)	39.3 (33.6, 45.4)
No	64.4 (60.1, 68.6)	67.6 (60.9, 73.7)	55.5 (50.4, 60.4)	39.5 (32.0, 47.5)	60.7 (54.6, 66.4)
**Years in the US/Nativity**, %***					
<10 Years	31.1 (27.0, 35.5)	39.3 (33.6, 45.4)	28.3 (23.3, 33.8)	22.3 (14.4, 32.9)	31.4 (26.5, 36.7)
≥10 Years	50.2 (45.9, 54.4)	45.2 (38.9, 51.7)	46.2 (41.1, 51.3)	38.9 (31.2, 47.2)	50.0 (44.5, 55.5)
US-Born	18.7 (15.1, 22.9)	26.6 (10.6, 22.0)	25.5 (21.3, 30.3)	38.8 (32.2, 45.8)	18.6 (13.9, 24.4)
**Ideal Cardiovascular Health (CVH)**					
Total Score, %	20.8 (17.8, 24.0)	24.1 (18.9, 30.1)	17.2 (13.9, 21.2)	22.1 (15.3, 25.9)	20.1 (15.3, 25.9)
**Ideal CVH Metrics**					
Blood Pressure, %	46.1 (41.8, 50.5)	46.0 (39.5, 52.6)	45.1 (40.0, 50.3)	40.9 (34.7, 47.5)	39.4 (33.4, 45.8)
Cholesterol, %	56.8 (52.7, 60.8)	53.9 (47.1, 60.6)	49.9 (44.7, 55.1)	48.6 (41.7, 55.5)	50.8 (45.1, 56.4)
Fasting Glucose, %	64.3 (59.9, 68.5)	67.7 (61.6, 73.3)	61.1 (56.4, 65.6)	70.3 (63.3, 76.4)	69.5 (63.9, 74.6)
BMI, %	23.0 (19.7, 26.6)	26.2 (21.2, 31.9)	18.2 (14.4, 22.7)	20.4 (15.5, 26.2)	21.8 (17.2, 27.1)
(non-) Smoking, %*	70.2 (65.7, 74.4)	72.3 (65.8, 77.9)	76.6 (71.9, 80.7)	83.1 (77.0, 87.9)	75.0 (69.7, 79.7)
Physical Activity, %	81.9 (78.6, 84.7)	80.8 (74.7, 85.7)	81.2 (76.6, 85.1)	81.5 (76.6, 85.6)	76.0 (70.8, 80.6)
Diet, %*	1.2 (0.7, 2.2)	1.1 (0.5, 2.4)	1.1 (0.5, 2.2)	1.5 (0.5, 4.9)	1.2 (0.5, 2.8)

^a^ Means and proportions are weighted (except sample sizes)

*p<0.05

**p<0.01

***p<0.001

Table 6 presents descriptive characteristics for employed females (only) according to employment type. Among females, the most common employment types were non-skilled workers (25.5%) and service workers (25.3%). Service workers were, on average, older than others (*p*<0.001). Professional workers had higher education (*p*<0.001) and annual household income (*p*<0.001) levels, compared to other employment types. Females who were professional workers had the highest unadjusted prevalence of ideal blood pressure (70.4%; *p*<0.05). There were no statistically significant differences in the unadjusted prevalence of ideal CVH score, ideal cholesterol, ideal fasting glucose, ideal BMI, ideal (non-) smoking, ideal physical activity, and ideal diet across employment types in females (Table 6).

**Table 6:** Descriptive characteristics by employment status among females (*n* = 3,718), HCHS/SOL (2008–2011).

	Non-Skilled Worker (*n* = 1,092)	Service Worker (*n* = 910)	Skilled Worker (*n* = 719)	Professional Worker (*n* = 500)	Other Worker (*n* = 497)
	Mean or Proportion (95% Confidence Interval)^a^				
Proportion	**25.5 (23.0, 28.1)**	**25.3 (22.8, 27.9)**	**19.3 (17.3, 21.4)**	**16.0 (14.0, 18.1)**	**14.0 (12.4, 15.9)**
**Age**, Mean***	38.4 (37.3, 39.6)	41.4 (40.1, 42.7)	38.6 (37.4, 39.8)	38.5 (37.0, 40.1)	36.8 (35.4, 38.3)
**Education**, %***					
<High School	40.4 (36.2, 44.6)	27.7 (23.1, 32.7)	17.7 (14.1, 21.9)	10.5 (5.4, 19.3)	21.6 (17.1, 26.9)
High School	32.0 (28.0, 36.2)	27.2 (23.3, 31.5)	19.4 (15.7, 23.6)	16.2 (11.8, 21.9)	31.5 (25.8, 37.7)
>High School	27.6 (23.8, 31.9)	45.1 (40.0, 50.2)	63.0 (57.3, 68.2)	73.3 (65.4, 79.9)	46.9 (40.8, 53.2)
**Annual Household income**, %***					
<$10,000	11.7 (9.0, 15.0)	12.4 (9.8, 15.7)	6.5 (4.6, 9.1)	2.0 (1.1, 3.5)	12.6 (9.1, 17.3)
$10,001–$20,000	36.0 (32.0, 40.1)	36.3 (32.3, 40.6)	25.6 (20.9, 31.1)	11.8 (8.9, 15.4)	33.7 (28.7, 39.0)
$20,001–$40,000	37.7 (32.9, 42.8)	29.0 (25.3, 33.1)	35.8 (31.1, 40.9)	42.9 (36.1, 50.0)	25.1 (20.4, 30.5)
$40,001–$75,000	8.6 (6.7, 11.1)	10.4 (7.5, 14.5)	20.5 (16.5, 25.3)	27.5 (21.9, 33.9)	17.2 (12.1, 23.9)
>$75,000	1.6 (0.9, 2.9)	2.0 (1.1, 3.7)	6.6 (4.2, 10.3)	13.9 (10.2, 18.7)	4.7 (2.9, 7.5)
Missing	4.3 (3.1, 6.1)	9.7 (7.3, 12.9)	4.9 (3.1, 7.6)	2.0 (1.1, 3.6)	6.8 (4.1, 11.1)
**Hispanic/Latino Background**, %***					
Cuban	4.6 (2.8, 7.5)	25.6 (20.9, 30.8)	11.5 (7.8, 16.5)	15.6 (11.4, 21.1)	12.7 (8.7, 18.1)
Dominican	10.4 (7.4, 14.3)	8.9 (6.4, 12.3)	17.9 (13.8, 22.9)	6.6 (4.0, 10.5)	14.2 (10.4, 19.2)
Mexican	61.5 (55.6, 67.1)	28.2 (22.8, 34.3)	46.2 (40.5, 52.0)	37.4 (31.0, 44.4)	41.4 (34.7, 14.8)
Puerto Rican	7.7 (5.8, 10.3)	5.9 (4.2, 8.4)	10.3 (8.0, 13.3)	18.0 (14.0, 22.9)	13.2 (9.5, 18.0)
Central American	8.6 (6.6, 11.1)	18.8 (14.9, 23.6)	3.9 (2.8, 5.4)	7.6 (5.1, 11.3)	7.1 (5.0, 9.9)
South American	4.9 (3.6, 6.7)	9.4 (7.4, 12.0)	3.0 (2.1, 4.3)	6.5 (4.1, 10.0)	7.0 (4.7, 10.4)
Other/Mixed	2.3 (1.3, 4.1)	3.1 (1.7, 5.5)	7.1 (4.5, 11.0)	8.2 (3.5, 18.2)	4.3 (2.3, 8.0)
**Current Health Insurance**, %***					
Yes	38.5 (34.0, 43.3)	40.8 (35.9, 45.8)	53.3 (47.6, 58.9)	67.4 (61.3, 73.1)	52.2 (46.0, 58.3)
No	61.5 (56.7, 66.0)	59.2 (54.2, 64.1)	46.7 (41.1, 52.4)	32.6 (26.9, 38.7)	47.8 (41.7, 54.0)
**Years in the US/Nativity**, %***					
<10 Years	28.8 (24.6, 33.4)	39.0 (34.3, 44.0)	25.2 (20.3, 30.9)	14.7 (10.7, 20.0)	30.7 (24.9, 37.1)
≥10 Years	54.6 (50.0, 59.1)	47.1 (42.1, 52.2)	49.4 (44.1, 54.7)	49.8 (43.3, 56.3)	43.8 (37.4, 50.5)
US-Born	16.6 (13.1, 20.8)	13.9 (10.5, 18.0)	25.4 (20.9, 30.4)	35.5 (29.6, 41.9)	25.5 (19.8, 32.1)
**Ideal Cardiovascular Health (CVH)**					
Total Score, %	31.5 (27.4, 35.8)	28.7 (24.8, 33.0)	28.2 (22.8, 34.2)	25.9 (20.7, 31.8)	27.6 (22.1, 33.8)
**Ideal CVH Metrics**					
Blood Pressure, %*	65.4 (61.0, 69.6)	60.2 (55.4, 64.7)	63.3 (61.1, 71.2)	70.4 (64.8, 75.5)	70.1 (64.8, 74.8)
Cholesterol, %	62.2 (58.1, 66.1)	56.9 (52.0, 61.7)	59.9 (54.7, 64.9)	55.9 (49.6, 62.1)	56.2 (49.6, 62.5)
Fasting Glucose, %	78.8 (75.3, 81.9)	74.8 (70.0, 79.1)	80.6 (75.2, 84.3)	80.9 (75.2, 85.6)	82.5 (78.2, 86.1)
BMI, %	22.1 (19.1, 25.4)	28.1 (24.3, 32.3)	26.7 (21.4, 32.7)	28.1 (22.4, 34.5)	25.5 (20.2, 31.7)
(non-) Smoking, %	87.3 (84.4, 89.7)	85.8 (82.8, 88.4)	85.8 (80.9, 89.6)	83.9 (76.1, 89.5)	82.8 (77.8, 86.9)
Physical Activity, %	65.0 (60.4, 69.4)	67.9 (63.5, 71.9)	63.2 (57.4, 68.6)	65.1 (59.2, 70.5)	67.7 (61.7, 73.1)
Diet, %	2.7 (1.5, 4.8)	1.8 (1.0, 3.4)	2.3 (1.3, 3.9)	0.6 (0.2, 1.7)	1.8 (0.6, 5.4)

^a^ Means and proportions are weighted (n’s are unweighted)

*p<0.05

**p<0.01

***p<0.001

Table 7 shows the sex-specific associations between employment type (with non-skilled worker serving as the referent category) and ideal CVH score and metrics. Among both males, and females there was no association between employment type and ideal CVH score. In analyses on the individual CVH metrics, males who were professional workers (vs. non-skilled workers) had an 8% higher prevalence of ideal (non-) smoking (*APD* = 8.2, *95% CI* = 1.2, 15.2). Females who were professional workers (vs. non-skilled workers) had an 8% higher prevalence of ideal blood pressure (*APD* = 8.4, *95% CI* = 2.5, 14.4) but also had a 2% lower prevalence of ideal diet (*APD* = -2.2, *95% CI* = -3.9, -0.5). Females in “other” occupations had a 9% lower prevalence of ideal cholesterol (*APD* = -8.6, *95% CI* = -15.9, -1.4) and females who were service workers had a 7% higher prevalence of ideal physical activity (*APD* = 6.8, *95% CI* = 0.1, 13.5).

**Table 7:** Associations between employment type and ideal cardiovascular health (CVH) among employed males (*n* = 3,209) and females (*n* = 3,718): Adjusted^†^ prevalence difference (APD) with 95% confidence interval (CI), HCHS/SOL (2008–2011).

	ALL AGES		MALES		FEMALES	
	Males (*n* = 3,209)	Females (*n* = 3,718)	Ages 18–44 (*n* = 1,635)	Ages 45–74 (*n* = 1,574)	Ages 18–44 (*n* = 1,650)	Ages 45–74 (*n* = 2,068)
APD (95% CI)						
**Ideal CVH**						
*Non-Skilled Worker (ref)*	*20*.*5 (17*.*2*, *23*.*8)*	*30*.*9 (26*.*9*, *34*.*9)*	*24*.*2 (20*.*0*, *28*.*5)*	*12*.*3 (8*.*4*, *16*.*3)*	*39*.*3 (34*.*0*, *44*.*6)*	*14*.*6 (9*.*6*, *19*.*6)*
Service Worker	4.3 (-2.1, 10.7)	2.0 (-3.5, 7.6)	5.2 (-3.5, 13.8)	0.5 (-6.3, 7.3)	5.8 (-2.4, 14.1)	-2.6 (-8.2, 3.0)
Skilled Worker	-3.0 (-7.8, 1.7)	-4.4 (-11.1, 2.3)	-2.2 (-8.8, 4.4)	-4.7 (-9.6, 0.2)	-4.9 (-14.5, 4.6)	-2.6 (-9.0, 3.8)
Professional	0.6 (-7.5, 8.7)	-6.6 (-14.0, 0.8)	3.4 (-7.4, 14.2)	-4.7 (-11.0, 1.6)	-9.0 (-18.8, 0.8)	-1.6 (-10.7, 7.5)
Other Occupation	-0.2 (-6.7, 6.3)	-5.8 (-13.3, 1.7)	-1.0 (-9.2, 7.3)	-0.1 (-6.8, 6.6)	-8.2 (-18.1, 1.6)	0.3 (-8.0, 8.6)
			*P*_*interaction*_ = 0.626		*P*_*interaction*_ = **0.027**	
**Ideal Blood Pressure**						
*Non-Skilled Worker (ref)*	*45*.*1 (40*.*8*, *49*.*4)*	*62*.*2 (58*.*2*, *66*.*2)*	*51*.*4 (45*.*8*, *56*.*9)*	*31*.*3 (26*.*1*, *36*.*6)*	*76*.*3 (70*.*8*, *81*.*8)*	*35*.*7 (29*.*9*,*41*.*6)*
Service Worker	1.4 (-6.7, 9.4)	3.5 (-2.4, 9.4)	3.9 (-7.4, 15.1)	-1.5 (-10.6, 7.6)	6.2 (-1.3, 13.7)	-0.1 (-9.2, 8.9)
Skilled Worker	0.7 (-5.8, 7.2)	3.4 (-3.0, 9.7)	2.4 (-5.7, 10.6)	-5.3 (-12.8, 2.1)	3.4 (-5.2, 11.9)	1.9 (-6.2, 9.9)
Professional	-3.8 (-12.7, 5.0)	**8.4 (2.5, 14.4)**	-5.4 (-16.6, 5.7)	-5.1 (-15.7, 5.5)	**8.5 (1.0, 16.0)**	5.4 (-5.7, 16.5)
Other Occupation	-5.4 (-12.8, 2.0)	4.5 (-1.3, 10.2)	-6.6 (-16.0, 2.7)	-2.0 (-10.8, 6.9)	5.7 (-1.9, 13.3)	3.9 (-4.9, 12.7)
			*P*_*interaction*_ = 0.298		*P*_*interaction*_ = 0.170	
**Ideal Cholesterol**						
*Non-Skilled Worker (ref)*	*55*.*9 (51*.*7*, *60*.*0)*	*60*.*7 (56*.*8*, *64*.*6)*	*60*.*4 (55*.*5*, *65*.*4)*	*46*.*9 (40*.*5*, *53*.*2)*	*72*.*8 (67*.*8*, *77*.*9)*	*38*.*1 (32*.*4*, *43*.*9)*
Service Worker	-1.4 (-9.3, 6.5)	1.3 (-4.8, 7.4)	-0.2 (-10.4, 10.0)	-9.5 (-19.3, 0.2)	5.9 (-1.8, 13.7)	-5.3 (-13.3, 2.7)
Skilled Worker	-4.9 (-11.0, 1.2)	-1.6 (-8.2, 4.9)	-2.0 (-9.4, 5.4)	**-12.1 (-21.4, -2.8)**	0.7 (-7.9, 9.4)	-7.6 (-16.0, 0.8)
Professional	-5.8 (-13.7, 2.1)	-6.1 (-13.5, 1.3)	0.0 (-10.0, 10.0)	**-17.9 (-30.3, -5.4)**	-4.4 (-13.8, 5.0)	**-12.4 (-21.8, -3.1)**
Other Occupation	-6.3 (-13.4, 0.8)	**-8.6 (-15.9, -1.4)**	-4.2 (-13.3, 5.0)	**-13.9 (-22.6, -5.1)**	-9.3 (-18.8, 0.3)	-6.3 (-15.5, 3.0)
			*P*_*interaction*_ = 0.279		*P*_*interaction*_ = 0.076	
**Ideal Fasting Glucose**						
*Non-Skilled Worker (ref)*	*64*.*0 (59*.*9*, *68*.*2)*	*80*.*8 (77*.*7*, *84*.*0)*	*69*.*8 (64*.*6*, *74*.*9)*	*50*.*8 (44*.*7*, *56*.*9)*	*88*.*8 (84*.*9*, *92*.*7)*	*65*.*5 (59*.*9*, *71*.*0)*
Service Worker	4.0 (-2.5, 10.6)	-5.1 (-11.2, 1.0)	5.8 (-2.5, 14.2)	-0.2 (-10.8, 10.4)	-5.4 (-12.5, 1.8)	-3.1 (-11.8, 5.7)
Skilled Worker	-2.2 (-8.0, 3.7)	-1.2 (-6.4, 3.9)	0.6 (-6.7, 7.9)	-7.7 (-16.7, 1.3)	-2.4 (-9.5, 4.6)	0.1 (-7.3, 7.5)
Professional	5.6 (-1.9, 13.0)	-1.7 (-8.0, 4.6)	8.4 (-0.4, 17.2)	-0.0 (-12.8, 12.8)	-3.3 (-10.7, 4.1)	0.6 (-9.7, 10.9)
Other Occupation	4.9 (-1.4, 11.3)	-0.5 (-5.5, 4.6)	7.1 (-1.3, 15.5)	-0.5 (-10.4, 9.4)	-1.5 (-7.6, 4.6)	1.9 (-7.4, 11.3)
			*P*_*interaction*_ = 0.670		*P*_*interaction*_ = 0.606	
**Ideal BMI**						
*Non-Skilled Worker (ref)*	*22*.*6 (19*.*3*, *26*.*0)*	*23*.*6 (20*.*2*, *27*.*1)*	*24*.*1 (19*.*6*, *28*.*5)*	*19*.*0 (14*.*1*, *23*.*8)*	*27*.*3 (22*.*6*, *32*.*1)*	*16*.*7 (12*.*5*, *21*.*0)*
Service Worker	3.1 (-3.6, 9.8)	6.0 (0.9, 11.1)	-1.4 (-10.0, 7.2)	8.5 (-0.4, 17.4)	7.0 (-0.7, 14.7)	2.9 (-3.4, 9.2)
Skilled Worker	-3.8 (-9.0, 1.4)	2.0 (-4.8, 8.9)	-2.3 (-9.3, 4.8)	-5.0 (-11.6, 1.6)	3.5 (-5.5, 12.6)	1.0 (-5.6, 7.6)
Professional	-1.2 (-8.1, 5.7)	2.1 (-4.6, 8.9)	1.3 (-7.9, 10.5)	-3.2 (-12.0, 5.6)	0.8 (-8.1, 9.7)	6.2 (-2.8, 15.3)
Other Occupation	-1.5 (-7.8, 4.8)	0.3 (-6.7, 7.2)	-0.9 (-9.0, 7.2)	-3.5 (-11.4, 4.4)	-2.2 (-11.1, 6.7)	4.9 (-3.3, 13.0)
			*P*_*interaction*_ = 0.506		*P*_*interaction*_ = 0.547	
**Ideal (non-) Smoking**						
*Non-Skilled Worker (ref)*	*71*.*5 (67*.*4*, *75*.*7)*	*87*.*1 (84*.*3*, *89*.*8)*	*71*.*2 (66*.*0*, *76*.*4)*	*71*.*2 (65*.*1*, *77*.*3)*	*87*.*7 (84*.*1*, *91*.*2)*	*85*.*5 (82*.*0*, *89*.*0)*
Service Worker	1.4 (-6.4, 9.2)	-1.0 (-4.8, 2.7)	1.9 (-0.8, 11.8)	1.2 (-7.9, 10.4)	1.0 (-4.0, 6.0)	-3.5 (-9.0, 2.0)
Skilled Worker	4.8 (-1.4, 11.0)	-1.8 (-6.9, 3.3)	4.7 (-3.5, 13.0)	6.7 (-1.1, 14.5)	-3.9 (-10.9, 3.2)	3.6 (-1.6, 8.8)
Professional	**8.2 (1.2, 15.2)**	-2.9 (-9.7, 4.0)	8.2 (-0.3, 16.7)	10.6 (-0.5, 21.8)	-5.2 (-13.8, 3.3)	2.5 (-3.8, 8.9)
Other Occupation	3.5 (-2.9, 9.8)	-3.7 (-8.9, 1.5)	2.1 (-6.3, 10.6)	7.4 (-1.1, 15.8)	-0.8 (-7.3, 5.6)	**-9.0 (-16.8, -1.1)**
			*P*_*interaction*_ = 0.970		*P*_*interaction*_ = **0.007**	
**Ideal Physical Activity**						
*Non-Skilled Worker (ref)*	*81*.*3 (78*.*3*, *84*.*3)*	*64*.*4 (59*.*8*, *69*.*0)*	*83*.*9 (80*.*1*, *87*.*6)*	*75*.*0 (69*.*1*, *81*.*0)*	*65*.*4 (59*.*3*, *71*.*5)*	*62*.*5 (56*.*4*, *68*.*5)*
Service Worker	0.3 (-5.6, 6.2)	**6.8 (0.1, 13.5)**	2.2 (-4.9, 9.3)	-3.5 (-13.2, 6.2)	8.7 (-0.4, 17.9)	3.9 (-4.9, 12.8)
Skilled Worker	-0.1 (-4.8, 4.7)	-2.3 (-9.6, 5.0)	1.8 (-4.2, 7.7)	-3.2 (-12.6, 6.2)	-2.1 (-12.1, 7.9)	-2.4 (-10.9, 6.1)
Professional	0.5 (-5.4, 6.5)	-1.9 (-9.4, 5.5)	2.6 (-4.6, 9.7)	-4.3 (-15.5, 7.0)	0.3 (-9.1, 9.8)	-6.0 (-16.6, 4.6)
Other Occupation	-5.1 (-10.5, 0.3)	2.9 (-4.4, 10.1)	-3.7 (-9.9, 2.4)	-7.3 (-17.2, 2.6)	0.3 (-9.3, 9.9)	8.6 (-0.3, 17.6)
			*P*_*interaction*_ = 0.916		*P*_*interaction*_ = 0.362	
**Ideal Diet**						
*Non-Skilled Worker (ref)*	*1*.*4 (0*.*5*, *2*.*2)*	*2*.*7 (1*.*3*, *4*.*2)*	*1*.*3 (0*.*2*, *2*.*4)*	*1*.*5 (0*.*5*, *2*.*5)*	*3*.*0 (0*.*9*, *5*.*1)*	*2*.*0 (0*.*7*, *3*.*3)*
Service Worker	-0.2 (-1.5, 1.2)	-0.8 (-2.6, 0.9)	-0.6 (-2.2, 1.0)	0.5 (-1.9, 2.8)	-1.8 (-4.0, 0.5)	1.0 (-1.3, 3.3)
Skilled Worker	-0.3 (-1.4, 0.8)	-0.5 (-2.4, 1.4)	-0.6 (-2.0, 0.7)	0.4 (-1.2, 2.1)	-1.9 (-4.4, 0.6)	2.6 (-0.8, 5.9)
Professional	-0.4 (-2.8, 2.0)	**-2.2 (-3.9, -0.5)**	0.2 (-2.8, 3.2)	-1.5 (-3.2, 0.3)	**-2.4 (-4.7, -0.1)**	-1.7 (-4.0, 0.6)
Other Occupation	**-**0.0 (-1.4, 1.3)	**-**0.9 (-3.3, 1.4)	**-**0.2 (-2.0, 1.6)	0.1 (-1.7, 1.9)	**-**0.8 (-4.0, 2.5)	**-**1.3 (-3.1, 0.4)
			*P*_*interaction*_ = 0.157		*P*_*interaction*_ = 0.120	

Results are weighted (n’s are unweighted).

^†^Adjusted for age (continuous), education, annual household income, Hispanic/Latino background, health insurance status, and years lived in the US/nativity.

Among females, age modified the associations of employment type with ideal CVH score and ideal (non-) smoking (*P*_*interaction*_ *=* 0.027 and 0.007, respectively). In age-stratified analyses, however, there were no associations between employment type and ideal CVH score in women aged 18–44 years or 45–74 years. Among females aged 45–74 years, those in “other” occupations had a 9% lower prevalence of ideal (non-) smoking than those who were non-skilled workers (*APD* = -9.0, *95% CI* = -16.8, -1.1). Among males, there was no evidence of effect modification by age in the association of employment type with ideal CVH score (Table 7).

## Discussion

The integration of health promotion efforts across the most influential day-to-day contexts such as workplaces, communities, and homes is critical for improving CVH at the population level and achieving AHA’s 2020 Impact Goal. This is the first study to examine the sex-specific associations between employment status and ideal CVH score and metrics in a population-based sample of Hispanic/Latino adults. In the present study, males employed FT (44%) and female homemakers (38%) represent the largest employment status groups in this population. As such, these groups are expected to make major contributions to the overall cardiovascular risk burden of Hispanics/Latinos and require special attention in CVH promotion efforts. Our findings provide an insight as to which CVH factors and behaviors could be targeted according to sex, employment status, and age.

Among males, we found that those who were employed PT had a higher prevalence of ideal CVH score but, among females, there was no association between employment type and ideal CVH score. Age-stratified analyses revealed various patterns in the prevalence of ideal CVH score among males. For instance, younger males who were employed PT had a higher prevalence of ideal CVH score, which could be due to the fact younger Hispanic/Latino males are more likely to be employed PT [44] and have a lower prevalence of ideal CVH than their older counterparts [9]. Middle-aged and older males who were homemakers or unemployed had a lower prevalence of ideal CVH score, which is consistent with previous studies showing that those who are unemployed [16–18] or homemakers [13,19,20] have a higher burden of CVD risk factors. Our findings are consistent with previous studies demonstrating the high burden of CVD risk factors in Hispanics/Latinos [45], and suggest that interventions designed to improve overall CVH among Hispanics/Latinos should target multiple settings and in particular, aim to promote ideal CVH among males who are homemakers and unemployed.

A better understanding of the associations between employment status and individual CVH metrics can also inform targeted efforts to improve CVH. We found that males who are employed PT, particularly younger adults, had a higher prevalence of ideal BMI than those who are employed FT. Strong positive correlations have been demonstrated between BMI and age [46,47], and between less than ideal BMI and adverse cardiovascular risk factors in this population [47]. We also found that males who are employed PT had a higher prevalence of ideal physical activity than those working FT. This finding suggests that Hispanic/Latino males who are employed FT may benefit from workplace interventions prioritizing promotion of healthy physical activity. The associations of employment with obesity and physical activity may be driven by the total number of hours worked [48,49], workplace sedentary time [50,51], and limited time for leisure time activities [51]. Those in urban workplaces may also have greater exposure to unhealthy commercial food environments which tend to be concentrated along commuting routes and workplaces [52].

Middle-aged and older females who were homemakers had a lower prevalence of ideal fasting glucose and ideal physical activity, compared to their employed FT counterparts. This observation is consistent with previous studies showing that middle-aged and older females who are homemaker (or unemployed) have worse CVD risk profiles than those employed FT [13–15]. However, to the best of our knowledge, there is no literature about health-promoting interventions tailored to Hispanic/Latina homemakers and many gaps in knowledge exist. For example, evidence about physical activity patterns among homemakers is limited. A previous analysis based on the National Health and Nutrition Examination Survey (NHANES) showed that in a multi-racial population-based sample of females aged 18–60 years homemakers were neither more sedentary nor less physically active than employed women [53]. It should be noted that the NHANES analysis was based on accelerometer data and ours was based on self-reported physical activity. The degree to which childrearing-related activities contribute to the overall energy expenditure among homemakers has not been well studied, even though incorporation of health promotion in this activity domain among females may increase the acceptability and uptake of the interventions. Lastly, while Hispanic/Latina females are commonly engaged in caregiving of older adults [54], it is not known whether such caregiving activities have a negative impact on health protective behaviors.

Our findings also highlight that that unemployed males and female homemakers had a lower prevalence of ideal (non-) smoking, compared to peers who were employed FT. Indeed, it has been shown that Hispanics/Latinos are more likely to live in communities with a higher density of tobacco outlets [55] and higher tobacco product sales [56]. Community-based campaigns to reduce tobacco availability could benefit the unemployed and homemakers. We also found that males and females who were unemployed had a lower prevalence of ideal physical activity compared to females who were employed FT. In efforts to promote physical activity among the unemployed, it is important to understand both the availability of neighborhood health resources (e.g., community centers, gyms, etc.) as well as factors that affect individuals’ ability to access such facilities such as perceived safety and transportation [56], a significant issue among low-income Hispanic/Latino adults. Lastly, it is important to note that, although males who were unemployed had a lower prevalence of ideal physical activity they also had a higher prevalence of ideal BMI compared to males who were employed FT. Plausible explanations for this inconsistent finding include that physical activity was self-reported increasing chances of misclassification and there could be presence of residual confounding bias, even after adjusting for age since unemployed males were younger than their employed counterparts.

No associations between employment type and ideal CVH score or metrics were seen among males with the exception of higher prevalence of ideal (non-) smoking among professional workers compared to non-skilled workers. Among females, some significant associations between employment type and ideal CVH outcomes were noted. For example, female professionals had a higher prevalence of ideal blood pressure but had a lower prevalence of ideal diet; service workers had a higher prevalence of ideal physical activity; and other occupations had a lower prevalence of ideal cholesterol. As such, the associations of employment type and ideal CVH score and metrics among females were not uniform across higher status of employment categories compared to lower status categories. Our findings on the associations between employment type and ideal CVH score or metrics may reflect the overall lower socio-economic status of Hispanics/Latinos [57], which may have hindered our ability to find associations across employment types.

This study has several limitations. As this is a cross-sectional analysis, we are unable to rule out the possibility of health-associated selection into employment status such as the tendency of less healthy individuals to be unemployed. We tried to limit this possibility by excluding individuals with existing CVD, chronic kidney disease, and physical limitations as well as retired adults. Voluntariness of employment or control over employment could not be measured in this study. We were unable to identify the specific characteristics of employment status that are associated with cardiovascular risk. Finally, given that the prevalence of ideal diet in the target population is low these results should be interpreted with caution. The strength of the present study is that it generated empirical evidence that may inform future health prevention research and targeted health initiatives related to employment status in the Hispanic/Latino population. This is one of the few studies to examine multiple types of employment (as opposed to binary categories) and it is the first study to examine the association of employment status and type with the construct of CVH among Hispanic/Latino adults from diverse backgrounds.

## Conclusions

According to the Social Determinants of Health framework [10,11], health promotion in workplaces and local communities is an important population-based approach to improve the CVH of Hispanics/Latinos. In the current study, we discovered sex-specific employment status-based differences in major CVH factors and behaviors among Hispanic/Latino adults. Importantly, the findings of this study draw attention to large “at risk” groups such as Hispanic/Latino who are homemakers or unemployed, and who have received little attention in the cardiovascular literature, and suggest the need for implementation of public health initiatives both in the workplace targeted towards Hispanic/Latino males who are employed FT and in communities targeted towards Hispanic/Latino males and females who are unemployed or homemakers.
